# A framework for increasing the availability of life cycle inventory data based on the role of multinational companies

**DOI:** 10.1007/s11367-017-1391-y

**Published:** 2017-10-04

**Authors:** Jamal Hussain Miah, Andrew Griffiths, Ryan McNeill, Sharla Halvorson, Urs Schenker, Namy Espinoza-Orias, Stephen Morse, Aidong Yang, Jhuma Sadhukhan

**Affiliations:** 1 Nestlé UK Ltd, Rowan Drive, Fawdon, Newcastle Upon Tyne, NE3 3TR UK; 2 Centre for Environment and Sustainability (CES), Faculty of Engineering and Physical Sciences, University of Surrey, Guildford, GU2 7XH UK; 3 Nestlé UK Ltd, Group Technical and Production, Haxby Road, York, YO91 1XY UK; 4 Nestlé Confectionery Product & Technology Centre (PTC), Haxby Road, York, YO91 1XY UK; 5 Nestlé Research Centre (NRC), CT-Nutrition, Health, Wellness and Sustainability, 1000 Lausanne 26, Switzerland; 6 Nestlé Research Centre (NRC), Sustainability & Novel Packaging, 1000 Lausanne 26, Switzerland; 7 Department of Engineering Science, University of Oxford, Parks Road, Oxford, OX1 3PJ UK

**Keywords:** Confectionery, Data collection, Food industry, Food products, Life cycle inventory, Multinational

## Abstract

**Purpose:**

The aim of the paper is to assess the role and effectiveness of a proposed novel strategy for Life Cycle Inventory (LCI) data collection in the food sector and associated supply chains. The study represents one of the first of its type and provides answers to some of the key questions regarding the data collection process developed, managed and implemented by a multinational food company across the supply chain.

**Methods:**

An integrated LCI data collection process for confectionery products was developed and implemented by Nestlé, a multinational food company. Some of the key features includes (1) management and implementation by a multinational food company; (2) types of roles to manage, provide and facilitate data exchange; (3) procedures to identify key products, suppliers and customers; (4) LCI questionnaire and cover letter and (5) data quality management based on the pedigree matrix. Overall, the combined features in an integrated framework provide a new way of thinking about the collection of LCI data from the perspective of a multinational food company.

**Results and discussion:**

The integrated LCI collection framework spanned across 5 months and resulted in 87 new LCI datasets for confectionery products from raw material, primary resource use, emission and waste release data collected from suppliers across 19 countries. The data collected was found to be of medium to high quality compared with secondary data. However, for retailers and waste service companies, only partially completed questionnaires were returned. Some of the key challenges encountered during the collection and creation of data included lack of experience, identifying key actors, communication and technical language, commercial compromise, confidentiality protection and complexity of multi-tiered supplier systems. A range of recommendations are proposed to reconcile these challenges which include standardisation of environmental data from suppliers, concise and targeted LCI questionnaires and visualising complexity through drawings.

**Conclusions:**

The integrated LCI data collection process and strategy has demonstrated the potential role of a multinational company to quickly engage and act as a strong enabler to unlock latent data for various aspects of the confectionery supply chain. Overall, it is recommended that the research findings serve as the foundations to transition towards a standardised procedure which can practically guide other multinational companies to considerably increase the availability of LCI data.

**Electronic supplementary material:**

The online version of this article (10.1007/s11367-017-1391-y) contains supplementary material, which is available to authorized users.

## Introduction

From the early days of life cycle assessment (LCA) over 40 years ago, the availability of Life Cycle Inventory (LCI) data has been a continuing major problem—a bottleneck—for the wide application of LCA (Testa et al. 2016; Ang et al. 2014; Finnveden et al. 2009; Pennington et al. 2007). As an internationally recognised and standardised approach, the application of LCA involves four phases which are (1) goal and scope definition, (2) inventory analysis, (3) impact assessment and (4) interpretation (ISO 2006). Overall, it is estimated that 70–80% of the time and cost involved in an LCA are related to data collection in the inventory phase by an organisation, especially for complex products that have several components and where the upstream and downstream supply chain structures are even more complex involving many actors (Testa et al. 2016; Ang et al. 2014; Berkhout and Howes 1997).

Since the advent of LCA, there are many published LCA studies where data collection is reported as a background activity (Resta et al. 2016; Meinrenken et al. 2014; Mila i Canals et al. 2011; Rebitzer and Buxmann 2005). The collection of data falls into two types: primary data and secondary data. Primary data are defined as “*directly measured or collected data representative of activities at a specific facility or set of facilities*” (European Commission 2013). For example, emissions/consumptions directly related to a specific process (Kim et al. 2015; Kellens et al. 2011), otherwise known as process LCI (Islam et al. 2016; Suh and Huppes 2005). Primary data tends to be highly specific and accurate. A variety of techniques can be used to collect primary data such as invoice bills, metered data, questionnaires, interviews and site visits (UNEP 2011; BSI PAS 2050 2011; European Commission 2010; EPA 1993, 1995, 2014). Once primary data is collected, the data is transformed into LCI for a range of environmental impacts such as Global Warming Potenital (GWP), ozone depletion and acidification (Bare 2011; Goedkoop et al. 2009; IPCC 2006; Guinée et al. 2002). In comparison, secondary data are defined as “*data that is not directly collected, measured, or estimated, but rather sourced from a third-party life-cycle-inventory database*” (European Commission 2013). This can also include data from publications and reports. However, secondary data tends to be less specific and highly aggregated. Some of the major LCI databases (DB) include Ecoinvent DB (Ecoinvent 2016), US LCI DB (NREL 2014), World Food LCA DB (WFLDB) (Nemecek et al. 2014) and Plastics Europe DB (PlasticsEurope 2015). For both primary and secondary data, there are guidelines available to ensure completeness, quality and transparency (Weidema et al. 2013; PEF World Forum 2013; UNEP 2011). Overall, for many LCAs, the common strategy for data collection is to collect the highest proportion of data from primary data sources which is carried out by an LCA practitioner. However, a considerable amount of time and cost is required by an LCA practitioner to physically collect primary data and rationalise and interpret LCI data as defined by the goal and scope of the LCA study (Testa et al. 2016; Jolliet et al. 2015; Ang et al. 2014).

In an effort to reduce cost and time of data collection, several approaches have been developed that streamline and simplify LCA methodology (Scanlon et al. 2013; Ning et al. 2013; Dowson et al. 2012) including reduction in LCA stages, e.g. gate-to-gate (factory) (Jimenez-Gonzalez et al. 2000); meta-product-based accounting (Mila i Canals et al. 2011); single impact categories, e.g. carbon dioxide or freshwater consumption (Stoessel et al. 2012); cut-off rules, e.g. 95% data coverage (Almeida et al. 2015); substitution of similar data (Dong et al. 2015) and simplification of the whole supply chain which are considered (Roches et al. 2010). However, despite these efforts, the availability of LCI data continues to be a consistent problem found in many LCA studies (Resta et al. 2016; Meinrenken et al. 2014; Mila i Canals et al. 2011).

Over the past 20 years, the primary and secondary data collected have been used to develop and populate LCI DBs dedicated at the national level, e.g. the US LCI (NREL 2014); Australian LCI (ALCAS 2011), Quebec LCI (Lesage and Samson 2016) and also at the sectorial level, e.g. WFLDB (Nemecek et al. 2014), Plastics Europe DB (PlasticsEurope 2015) and for agricultural products such as AgriBalyse DB in France (Koch and Salou 2013; Colomb et al. 2015) or Agrifootprint DB in the Netherlands (Agri-footprint gouda 2014). However, current LCI DBs are limited in available data that is current and of high quality. In addition, another aspect which is rarely discussed is the major gaps from the information in the public domain and available LCI datasets given the considerable rise in environmental reporting by companies across the full supply chain (Corporate Register 2017). Although such information may not be suitable as LCI data, what they do demonstrate is the potential available data and actors that can be harnessed to provide suitable data for LCA applications.

**Fig. 1:**
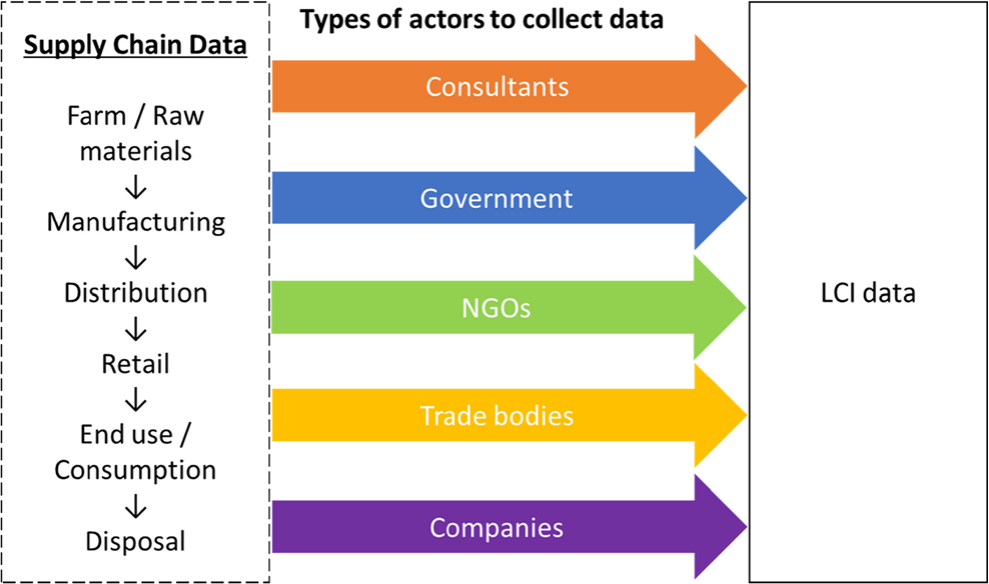
Different types of actors which can play a role to collect LCI data

Traditionally, the central vehicle to collect and compile LCI has been by consultants (Ecodesk 2015). However, the effectiveness of consultants to facilitate data exchange is limited as shown by the availability of data in current LCI DBs. As such, alternative strategies have emerged which involve single or multiple actors to catalyse participation and encourage cooperation across the supply chain to increase data availability, as shown in Fig. 1.

Due to the involvement of different actors, a range of different strategies have been developed to facilitate and collect LCI. For example, web-based systems (Ramos et al. 2016; BONSAI 2016; Recchioni et al. 2015; Mistry et al. 2016; Bellon-Maurel et al. 2014), trade bodies/industry associations (Jungbluth et al. 2016; Popp et al. 2013; Finkbeiner et al. 2003; Pomper 1998) and consultants (Credit 360 2015; Ecodesk 2015). However, the collection of data by these routes requires the strong involvement of actors across the whole supply chain where the main strategy and implementation process in terms of collecting data and data quality checks has been on a voluntary basis, promoted and instigated at a top level by a third party, e.g. research institutes, universities, governments, industry associations and consultants (Recchioni et al. 2015; Skone and Curran 2005). Even so, the ability of a third party to effectively engage and therefore collect data in a reasonable and practical timeframe with actors across the supply chain will be limited as they will not have full knowledge of the supply chain or the limitations of internal processes adopted by actors across that chain (Lesage and Samson 2016).

Another strategy that has received little attention is a company-led approach, especially from the perspective of powerful and influential actors such as manufacturing and retail companies. This is an important, and perhaps surprising, gap in the literature as due to the integration of manufacturing and retail companies within supply chains, they offer the opportunities to engage, initiate, collect, influence and manage LCI data directly through actors across the supply chain. As such, our hypothesis is that a company-led approach to data collection can provide an effective means to collect data. In order to satisfy this hypothesis, this paper seeks to address several research questions by presenting an effective and novel LCI data collection process and the implementation experience by Nestlé, a multinational food company for confectionery products. The research questions are as follows:

1. What is the timeframe to collect inlet/outlet flow data and can it be accelerated?
2. How much data should be collected and are their limitations on quality?
3. What are the effective tools to collect data?
4. Who are the key actors in the supply chain and how to identify them?
5. How effective is a company acting as the facilitator for data exchange?
6. What are the motivations for data exchange?
7. What are the challenges of collecting LCI data?
8. Can the collection of inlet/outlet flow data be standardised?
9. What is the resource required to collect inlet/outlet flow data?
10. What are the quality controls required to ensure robust datasets?
11. What company initiatives are recommended to promote an efficient LCI data collection?

The paper begins by presenting the proposed LCI data collection process employed by Nestlé in Sect. 2. This is followed by a selection of results of the LCI data collection process for confectionery products in Sect. 3. A discussion of the implementation experience, key challenges encountered and how the Nestlé LCI process compares to other initiatives and—in particular—what were the major differences and what we can learn from Nestlé’s experience that will help with LCI collection is provided in Sect. 4. Lastly, the conclusions are provided in Sect. 5.

## Methods

### Description of case company and food factory

The case company is Nestlé UK Ltd., a large food company in the UK and a subsidiary of Nestlé SA who are a global leading nutrition, health and wellness food company. Across the globe, Nestlé are active on addressing many sustainability issues related to the Sustainable Development Goals (SDGs) as part of their Creating Shared Value (CSV) strategy (Nestlé 2015a). For example, working with smallholder farmers through the Nestlé Cocoa plan (Nestlé 2015b) and Nestlé Nescafe plan (Nestlé 2015c), assessing and optimising the environmental impact of Nestlé products by LCA-based approaches (Nestlé 2013) and contributing to the development of environmental data across the supply chain such as the World Food LCA database (WFLDB 2014). As an organisation, there is not only the potential but a broad array of experience which can contribute to supply chain engagement and expedite data collection across the supply chain.

In the UK, Nestlé have 14 food factories that manufacture a range of products that include coffee, cereals, pet food, water and confectionery. The case factory is based in the North East of England that manufactures a range of confectionery products that are sugar, chocolate and biscuit based by utilising a diverse range of processing technologies. In total, there are approximately 130 Stock Keeping Units (SKUs) which are a variation of a brand product format, e.g. single bar pack and multiple bars pack. The SKUs are sold to a range of customers both in the UK and across the globe (Miah et al. 2015a). The use of a case study in this way allows for an in-depth exploration of the supply chain, and while it is acknowledged that the findings are specific to that chain, it can be reasonably surmised that the results are applicable for other multinational food companies who manufacture and sell food products directly to retailers.

### Overview of confectionery LCI data collection process

**Fig. 2:**
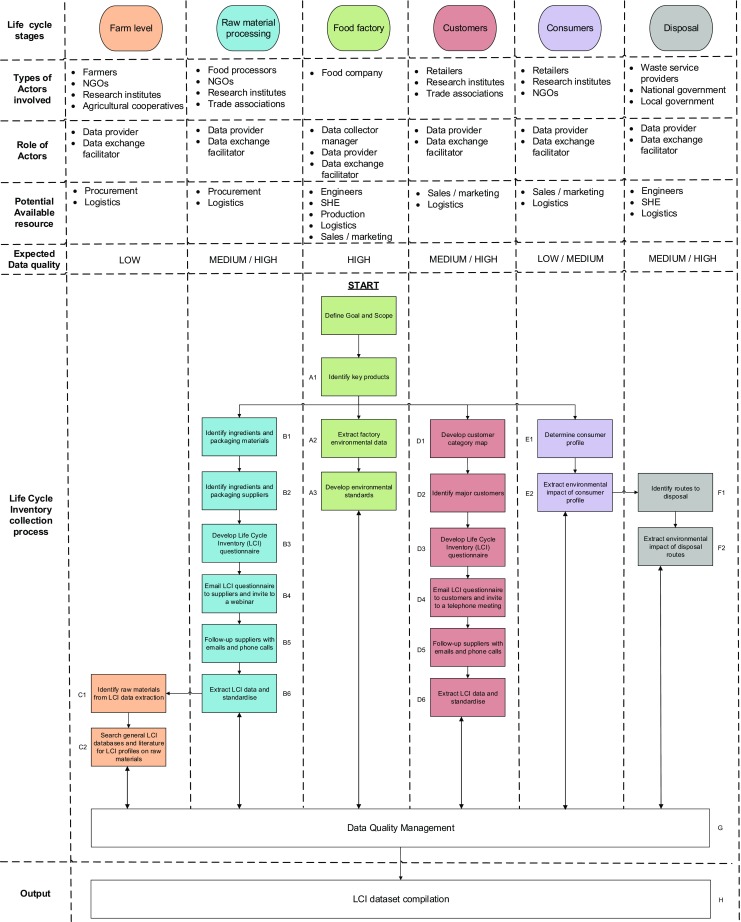
Life Cycle Inventory (LCI) data collection process

The LCI data collection process was initiated and developed by a transdisciplinary process involving both Nestlé practitioners and academics from the University of Surrey (Miah et al. 2015b). The LCI data collection process presented here (Fig. 2) is based on LCI guidelines (Nemecek et al. 2014; ALCAS 2014; UNEP 2011; BSI PAS 2050 2011; European Commission 2010) and the challenges faced by Rebitzer et al. (2004) and Berkhout and Howes (1997). As a methodology, the LCI data collection process displays features which are found in approaches by different companies, e.g. data sources, questionnaires, data quality management, etc. What distinguishes the approach presented here is the combined features and, more importantly, the role of a multinational food company (e.g. Nestlé), rather than a third party, to initiate, motivate, accelerate and manage the whole collection of inlet/outlet flow data across the supply chain.

The goal of the LCI data collection process is to provide an effective and efficient streamlined route to practically collect data—on a voluntary basis—across different input intensities such as electricity, natural gas, water and solid waste that is both specific and general at different stages of the product supply chain that can be used to conduct an LCA, e.g. environmental hotspot analysis.

The scope of the primary data collection process includes first-tier suppliers, factory, retailer, consumer and disposal. The farm-level stage was not included due to the indirect relationship with farmers and existing Nestlé initiatives such as the Cocoa plan (Nestlé 2015b), Nescafe plan (Nestlé 2015c) and contributing partner to the World Food LCA database (WFLDB 2014). The integrated LCI data collection process begins at the food factory because food manufacturers typically carry out the design of the product which sets forth the product supply chain structure both upstream and downstream. From here onwards, the data collection strategy branches both upstream and downstream of the product supply chain where the collected data is reviewed, analysed and normalised, if required. The final stage involves a reconciliation and aggregation of LCI datasets.

The responsibility for the whole management and implementation (including analysis) of the LCI data collection process is by a single person in Nestlé known as the ‘data collector manager’. On occasion, internal and external LCA experts are sought for advice. Overall, a range of people are involved throughout whose role falls into two categories: (1) data provider and (2) data exchange facilitator. The ‘data provider’ are people from different organisations across the stages of the life of a food product which provide data. The ‘data exchange facilitator’ are people primarily from Nestlé who have established relationships with data provider organisations to facilitate data exchange. From Nestlé’s perspective, an indicative level of resource required and expected data quality is provided at each life cycle stage as guidance. The different stages are explained in the following subsections.

### Description of the potential available resource

The potential available resource is an indication of the different people that could potentially be made available from the food company to participate in the collection of inlet/outlet flow data. The process to identify people is a continuing process but starts during the goal and scope definition, before the identification of SKUs, by developing a list/map of potential available resource based on recommendations from the decision-maker who commissioned the LCA. The decision-maker is likely to be someone in a senior position responsible for environmental sustainability improvements in the company. Following this, further people can be identified as data collection progresses. The types of people involved are primarily internal to the food company from the environment/sustainability department to provide further guidance and direction towards data providers both internal and external to the food company. For example, at the factory life cycle stage, the food company is directly involved with the management and operation of the food factory and will have several departments where various data is collected related to the environment. As such, there are a large number of people that could be coordinated to collect inlet/outlet flow data at the factory life cycle stage. In comparison to the farm-level life cycle stage, the food company will not necessarily have a direct involvement with the management and operation of the farm as Nestlé does not own farms. Although, they do have direct suppliers, where a strong relationship is established, through which data collection is possible indirectly to the farmers. As such, there will be a low number of people that could be coordinated to collect inlet/outlet flow data at the farm-level life cycle stage. Overall, the types of people involved internally to the food company will vary depending on the life cycle stage as different departments or functions will have varying knowledge based on their role, experience and the relationships they have with people both internally and externally via institutions. The degree of engagement of human resources in LCI-related activities will vary for different food companies, but a general description is provided in Table 1 to distinguish between low, medium and high resources. The direct relationship refers to a business/professional relationship. On the other hand, the indirect relationship refers to the business/professional relationship with an intermediary to collect data from the life cycle stage.

**Table 1:** The degree of engagement of human resources in LCI related activities

Human resources	Description
Low	• No involvement in the life cycle stage • Indirect relationship with life cycle stage operator via an intermediary, e.g. co-operatives
Medium	• No direct involvement in the life cycle stage management or operation • A mix of direct and indirect relationships with life cycle stage operator
High	• A direct involvement in the life cycle stage via management and/or operation • A range of departments actively involved in environmental issues

### Description of data quality management

The management of data quality primarily involves the validation of data from the various life cycle stages to ensure data is robust, and thereby reduces the level of uncertainty in further analysis. A semi-quantitative assessment method known as the pedigree matrix is used which was originally developed by Weidema and Wesnaes (1996) and has gained traction over the course of 20 years to become the de facto quality assessment method for several LCI DBs (Ecoinvent 2016; NREL 2014; ALCAS 2011). The pedigree matrix contains ratings for different data quality indicators (DQIs) such as reliability (R), completeness (C), temporal correlation (TC), geographical correlation (GC) and technological correlation (TeC). The DQIs are then assessed based on the judgement of experts (e.g. LCA practitioners) and converted into a data quality score (DQS) by Eq. (1). The score is rated into high (DQS ≤ 1.6), medium (DQS ≥ 1.6 to <3) and low (DQS ≥ 3 to ≤ 5) quality.

1 $$ DQS=\frac{R+C+ TC+ GC+ TeC+{X}_W\times 4}{i+4} $$

where

DQS: data quality score

R, C, TC, GC, TeC: see values found in Weidema and Wesnaes (1996)

X_W_: weakest quality level obtained (i.e. highest numerical value) among the data quality indicators
*i*: number of applicable data quality indicators

The data quality management process involves reviewing the data provided to (1) screen for any data gaps, (2) identify anomalies in datasets and (3) ascertain data quality as described in Weidema and Wesnaes (1996) and Eq. (1). Based on the review, a list of questions is developed and sent to the data provider for clarification. From here onwards, a two-way dialogue (via emails, phone calls and physical meetings) continues with the aim to increase the quality of data to the highest quality level which is practical and economical to collect. Overall, throughout the data analysis approach, internal and external LCA experts are sought to provide additional quality assurances on the compiled dataset. For example, possible explanations of anomalies in data and verification of expected results.

### Description of food factory data collection: stages A1–A3

After the goal and scope was defined, the next step was to identify key products which can include distinct product categories and major products. The identification process was carried out through engagement with the factory production team who were able to provide production data split out into product categories. For the list of SKUs in each key product category, the major SKU was selected based on a Pareto analysis of the SKU production volumes which can be extracted from production and sales records. The major SKU is thus the reference product for the key product category throughout the whole LCI data collection process.

At a factory level, the input intensity monitored will typically cover energy, water, solid waste and liquid waste. The scale of available data will vary depending on the coverage of utility meters across site and within processes, billed utility invoices and systems to record physical materials, e.g. solid waste. As such, a combination of the available data in conjunction with reasonable estimates based on expert judgement was needed to allocate the input intensity down to a key product group based on mass allocation. A general rule for the allocation process is not possible as this will depend on the combination of available data and expert judgement. Alternatively, an economic allocation approach can be used if economic data is readily available. However, the major limitation compared to a mass allocation approach is the representation of input-output flows based on economic data rather than physical dimensions based on mass; hence, this is subject to price variability. As such, an economic allocation is recommended when mass data is not available.

### Description of raw material processing data collection: stages B1–B6

For the major SKU identified, a list of ingredients and packaging materials was determined based on the product recipe and packaging specification. The source of the data was obtained from the production specialists at the food factory. Following this, the identification of suppliers involved engaging with the procurement team of the food manufacturing company who has a business relationship with the suppliers and is able to formally and more appropriately request information. Prior to contacting the suppliers, an LCI questionnaire and cover letter was developed to provide the suppliers with the motivations of the request and the types of information required. The design of the questionnaire contains a range of information categories shown in Table 2. The questionnaire template can be found in the Electronic Supplementary Material.

**Table 2:** An overview of LCI questionnaire categories and general content

Information category	Description of content	Data provider
Supplier/customer overview	• Basic information on supplier to include material names and manufacturing site locations. • Confirmation of environmental management systems • Confirmation of previous LCAs in the company • Confirmation of willingness to collect further data down the supply chain	Supplier
Production	Production volumes of the factory and raw materials required to manufacture ingredients/packaging	Suppliers
Land footprint	The area space occupied by the total site and factory	Suppliers and customers
Store volume	The volume space occupied by the retail site and/or warehouse	Customers
Energy	Includes the different energy types: electricity, natural gas, fuel oil consumed at a factory and if possible at a product level	Suppliers and customers
Water	Includes the different water types: main, ground, river, and recycled consumed at a factory and if possible at a product level	Suppliers and customers
Atmospheric emissions	Includes the release of pollutants (if measured) to the atmosphere including particulate matter	Suppliers
Solid waste	Includes solid materials that are discarded off-site and not recycled on-site	Suppliers and customers
Liquid waste	Includes liquid process water sent to a wastewater treatment plant and any liquids that are siphoned into tanks to be treated off-site	Suppliers
Transportation	Includes a general breakdown of the transportation route from the location of manufacturing to the customer location	Suppliers and Customers

The cover letter developed was contained to a single page to keep the communication concise. It included the purpose of the data request, contact details and a deadline of 4 weeks from receipt. The cover letter was signed off by the procurement contact who managed the business relationship with suppliers and by the head of sustainability and head of procurement in the food manufacturing company. This was to ensure the request was supported at a high level in the food manufacturing company.

Both the inlet/outlet flow questionnaire and cover letter were sent via e-mail to the business contact in the supplier company. The option to follow-up with a webinar or phone call was provided. Any further communications took place through e-mails to discuss and clarify the request in more detail. When the inlet/outlet flow questionnaire was returned, the data were reviewed to gauge the sensibility of the information. The LCI data for each inlet/outlet flow obtained for a specific geographic location were then converted to a range of life cycle impacts per tonne of material manufactured based on different life cycle impact assessment (LCIA) methodologies discussed in Sadhukhan et al. (2014) using GaBi 6.0.

### Description of farm level data collection: stages C1–C2

The raw materials required to manufacture the ingredients and packaging materials can be found in the information extracted from the inlet/outlet flow questionnaires sent to the food manufacturing company suppliers. For the raw materials that could not be extracted or were not available due to incomplete or unreturned inlet/outlet flow questionnaires, literature searches were carried out on the general manufacture of ingredients and packaging materials to create a list of raw materials. Once a list of raw materials was made, they were categorised into similar groups (e.g. dairy includes milk, whey etc) adopting the approach by Mila i Canals et al. (2011) for modular ‘builidng blocks’. Afterwards, the raw material groups were cross-referenced with commercial LCI databases to find similar LCI profiles (Ecoinvent 2016; Quantis 2014; ALCAS 2011).

### Description of customers’ data collection: stages D1–D6

The portfolio of customers for confectionery products can be highly diverse depending on how developed the market where the products are sold, e.g. high street retailers to convenience stores to cinema outlets to snacks on an aeroplane. As such, the development of the range of customer categories was based on the literature (Spencer and Kneebone 2007; Spencer and Kneebone 2012) and from the food manufacturing company logistics team. For the major SKUs, it was possible to extract delivery orders over a 1-year time period to identify major customer categories and specifically the customers per se. From the identification of the major customer, it was possible to identify the key account manager inside the food manufacturing company who manages the business relationship with an equivalent in the customer company. At the same time, the sustainability/environmental contact in the food manufacturing company was able to provide an equivalent contact in the major customer category from previous and ongoing relationships. Before contacting the customer, a tailored LCI questionnaire was developed to include the same information categories as for customers. The processing of the returned inlet/outlet flow questionnaire is the same as discussed in Sect. 2.6.

### Description of consumers’ data collection: stages E1–E2

As most confectionery products are ‘ready to eat’, they do have short shelf-lives, and consumers are not expected to carry out any processes before consuming them. In this particular scenario, consumer behaviour regarding transportation from point of purchase (customer store location) to consumption, storage of product, food waste and disposal of packaging are the relevant parameters to be evaluated. Therefore, the process to collect data for the major SKUs was largely based on literature supported by the marketing and sales team in the food manufacturing company and retailers.

### Description of disposal data collection: stages F1–F2

For the major SKUs and based on the consumption behaviour of the consumer, the waste materials can be identified. The process to determine the routes to disposal should in principle follow the waste hierarchy (European Commission 2008), but in practice, this can differ where there are national averages that can be taken that provide recycling rates and disposal to landfill (EA 2014). For more specific environmental impact of different waste treatment options, the engagement with waste service providers that operate on a local or national level can provide data on a kilogram basis.

## Results

### Amount of data collected

The amount of data collected from both primary and secondary data sources are shown in Table 3. Overall, 183 LCI datasets were targeted for specific ingredients of which 129 were collected from primary and secondary sources. The total primary data collected was 100 whereas secondary data represented 29.

**Table 3:** Amount of LCI datasets collected from both primary and secondary data sources

	Life cycle stages						
	Farm/raw materials (C1–C2)	Raw material processing (B1–B6)	Factory (A1–A3)	Distribution (D1–D6)	Retail (D1–D6)	Use (E1–E2)	Disposal (F1–F2)
Target amount of LCI datasets	22	147	4	1	1	n/a	8
Total number of primary LCI datasets collected	0	96	4	0	0	n/a	0
Total number of secondary LCI datasets collected	13	0	0	1	1	n/a	8
Total number of no data collected	9	51	0	0	0	n/a	0
Percentage of total primary data collected (%)	0%	65%	100%	0%	0%	n/a	0%
Percentage of total secondary data collected (%)	59%	0%	0%	100%	100%	n/a	100%
Percentage of no data collected (%)	41%	35%	0%	0%	0%	n/a	0%

### Types of data collected

A range of primary data was collected for the factory, raw material processing and retailer shown in Tables 4 and 5 and Electronic Supplementary Material. For the conversion of primary data to environmental impacts, this was based on the energy data collected. The collection of emissions data was not found to be available across the majority of data providers as this was not measured and/or was confidential.

**Table 4:** Factory and product category level environmental resource consumption

Scale	Number of SKUs	Electricity (kWh/ton)	Natural gas (kWh/ton)	Water (m^3^/ton)	Solid waste (ton/ton)
Confectionery factory	130	539	1045	3.55	0.041
Chocolate product category	9^a^	412^b^	701^b^	4.16^b^	0.020^b^
Sugar product category	8^a^	642^b^	1570^b^	3.64^b^	0.057^b^
Chocolate biscuit product category	3^a^	714^b^	1081^b^	2.22^b^	0.070^b^

^a^Major SKUs

^b^Estimated based on average SKU

**Table 5:** Environmental aspects of retail in ambient conditions, for different scales

Scale	Electricity (kWh/m^2^ day)	Natural gas (kWh/m^2^ day)	Water (m^3^/m^2^ day)	Solid waste (ton/m^2^ day)
Superstore	0.0944	0.0267	1.53 × 10^−4^	6.02 × 10^−6^
Supermarket	0.0419	0.0113	7.12 × 10^−5^	5.21 × 10^−6^
Warehouse	0.021	0.00877	8.77 × 10^−5^	1.42 × 10^−5^

For the confectionery factory, the input intensity data is provided at the factory and product category level, shown in Table 4. Overall, the sugar product category has the highest natural resources consumption.

The LCI questionnaire and cover letter developed were sent via e-mail to 67 ingredients and packaging suppliers requesting 2013 data only. In total, only 55% returned questionnaires that went through a review process with the suppliers over a series of e-mails before being converted on a relative basis, e.g. per ton of bulk product delivered to the confectionery factory. The LCI data were then converted to a range of environmental impacts to widen the application depending on the preference of LCA practitioner, see Electronic Supplementary Material for full LCIA data.

Similar to raw material processing, an LCI questionnaire and cover letter was sent to two major food retailers in the UK. However, only one retailer was able to provide some information which was not in the correct format, as shown in Table 5.

### Quality of data collected

#### DQSs for both primary and secondary data

**Fig. 3:**
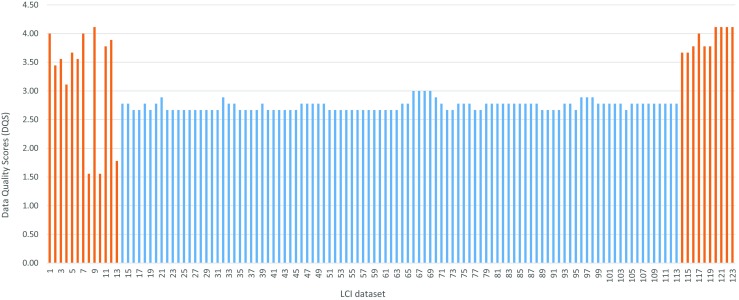
A comparison the DQS for 129 LCI datasets collected

The data collected was assessed based on the pedigree data quality matrix. A comparison of the calculated data quality score (DQS) for 123 LCI datasets is shown in Fig. 3. The orange bars represent secondary data whereas the blue bars represent primary data.

Overall, the DQS were then categorised into high-, medium- and low-quality groups, as shown in Table 6.

**Table 6:** DQSs for data collected categorised into high, medium and low data quality

Data quality group	Farm	Raw materials processing	Factory	Distribution	Retail	Disposal
High	2	0	0	0	0	0
Medium	1	96	4	0	0	0
Low	10	0	0	1	1	8

For the DQS, two statistical analysis techniques are used to determine variability by calculating the average and standard deviation, as shown in Table 7. The average DQS shows that the raw materials processing data has on average the best quality compared to data collected for the other life cycle stages. However, caution must be taken in the interpretation as the sample size for the different life cycle stages are considerably different and will influence the final results. Despite this, the rank of highest to lowest quality based on the average is raw material processing, factory, farm and disposal. Furthermore, when investigating the variability of data within each life cycle stage, the calculated standard deviation shows the factory has the lowest variability whereas the farm stage has the highest. The rank of lowest to highest variability based on the standard deviation is factory, raw material processing, disposal and farm. Overall, the statistical analysis shows the primary data collected for the factory and raw material processing has the highest quality.

**Table 7:** Statistical analysis of DQS

	Farm	Raw materials processing	Factory	Distribution^a^	Retail^a^	Disposal
Average	3.23	2.74	2.78	n/a	n/a	3.97
Standard deviation	0.95	0.08	Negligible	n/a	n/a	0.17

^a^One dataset only available

### Effectiveness of tools and processes deployed

**Fig. 4:**
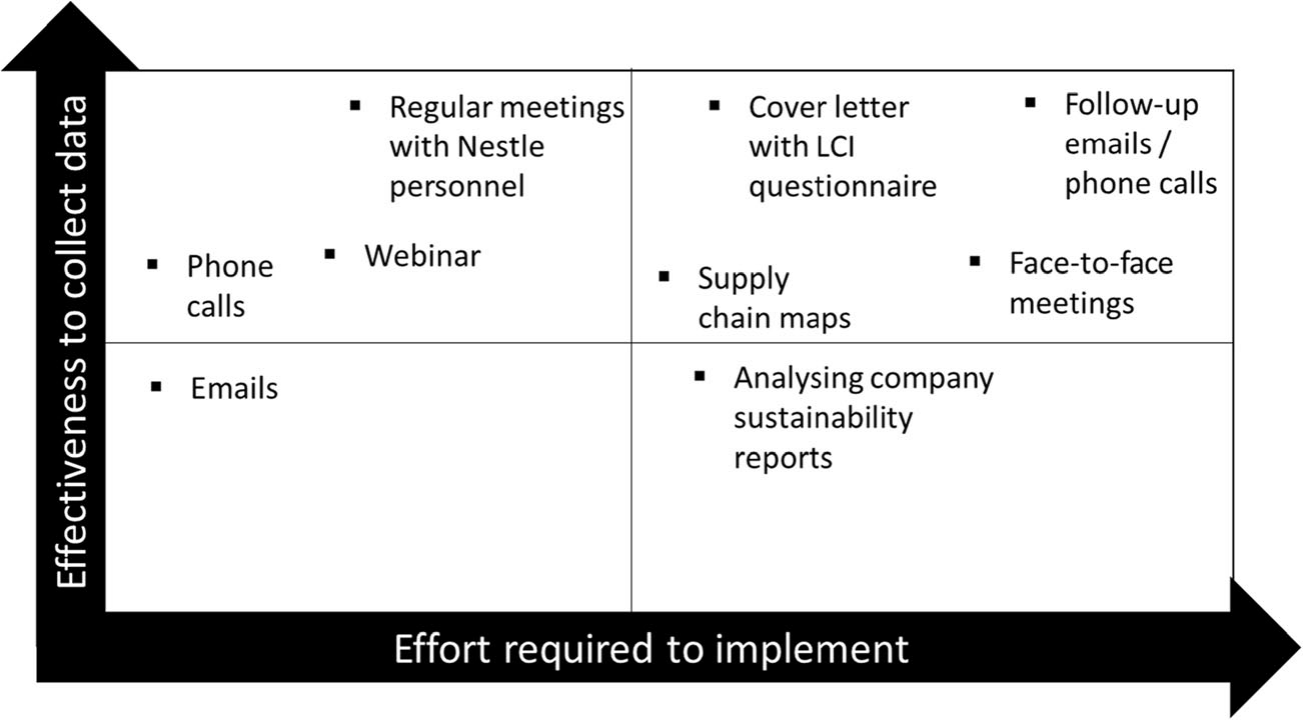
Assessment of the effectiveness of tools deployed

A subjective assessment is made of the tools and processes deployed through the data collection process in terms of the effectiveness to collect data and effort required to implement, as shown in Fig. 4. A comparison of the effectiveness of tools and processes are discussed in Sect. 4.

**Fig. 5:**
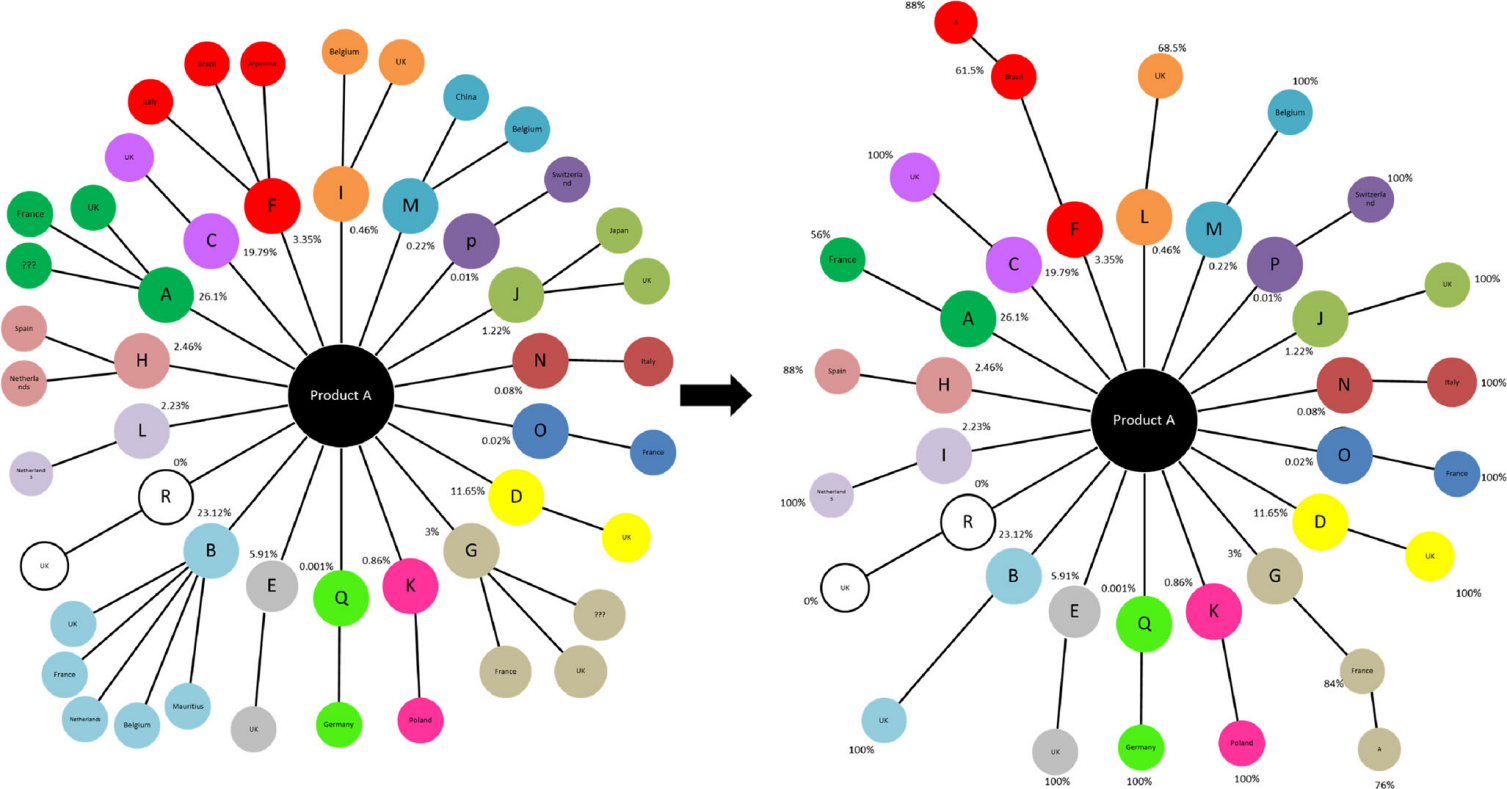
Two ingredients map showing how suppliers were reduced from start to end

A range of visual diagrams were also created which improved understanding of different aspects of the confectionery supply chain. For example, the identification of raw materials and their associated suppliers was strongly aided by the development of an ingredient map for 20 major SKUs (see Fig. 5 for one SKU). In Fig. 5, the inner circle represents ingredients (coded from A to R), and the percentages shown are the share of their contribution to material supply. The outer circle shows the origins of the materials. Initially, the maps were generated for all suppliers for each ingredient but over the course of time, they were narrowed down to single supplier for each ingredient based on highest percentage procured. In total, 147 ingredients and packaging materials purchased from 67 suppliers in 19 countries were identified.

**Fig. 6:**
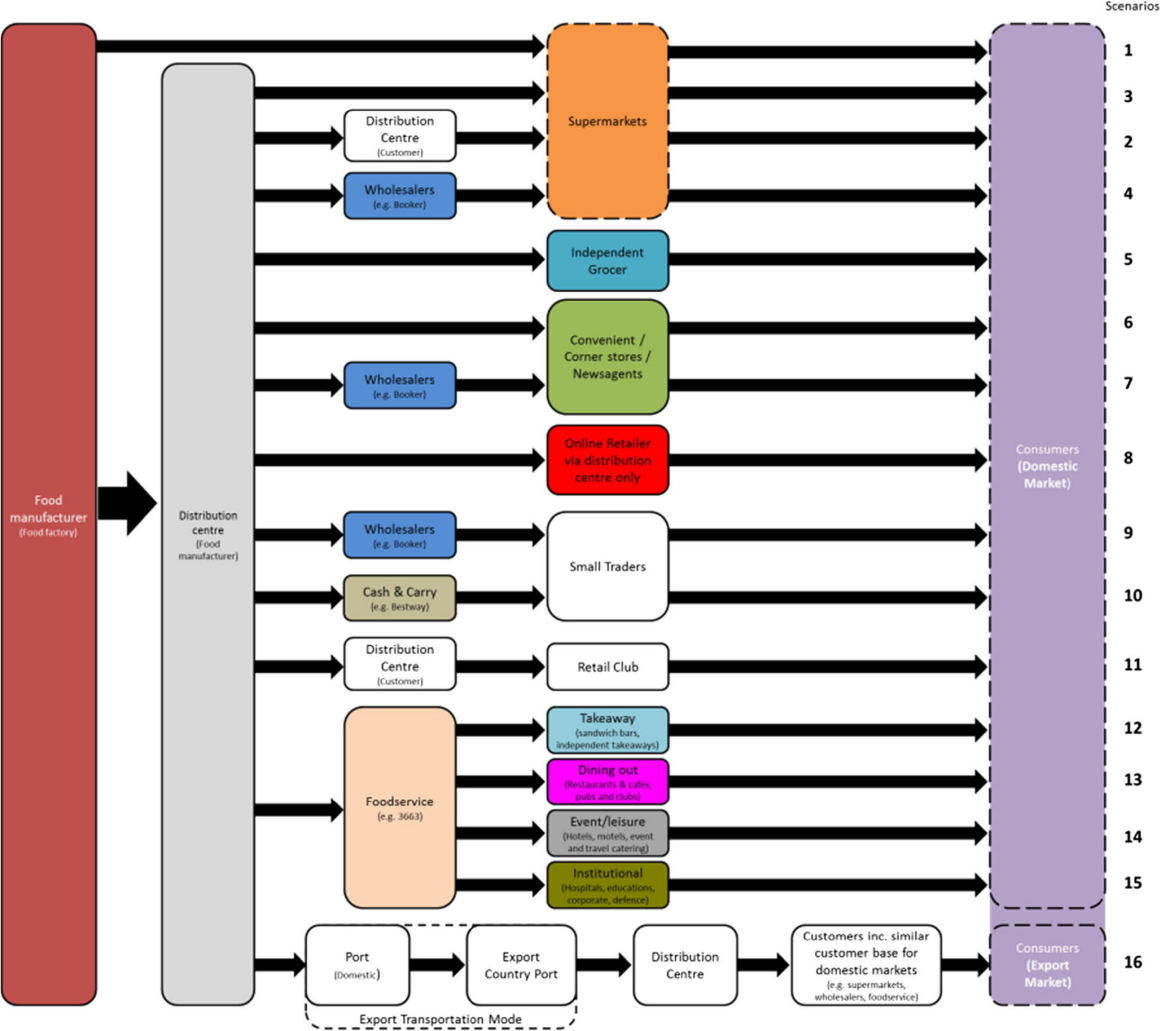
Customer distribution channels and customer categories for confectionery products

Another aspect of the supply chain which was visually represented was the upstream confectionery supply chain in terms of distribution and retail. Due to the history of Nestlé in the UK, they have developed mature channels to a range of customers in the UK. As such, the upstream section of the supply chain is complex and diverse. Initially, the starting point to collect data was from retailers but it was unclear if this was the right choice given the diversity of customers. In order to navigate through the complexity, a literature review was carried out to find different distribution channels for food products. The information collected was combined with Nestlé data based on discussions with the marketing and sales teams to create a full and general representation of the entire customer portfolio for confectionery; see Fig. 6.

### Challenges encountered during primary data collection

In the course of the LCI data collection process, Nestlé encountered several challenges which are shown in Table 8 with a range of recommendations proposed to resolve.

**Table 8:** List of challenges encountered by Nestlé

Challenges	Recommendations
1. Lack of engagement from supply chain actors	1. Supplier engagement events to raise awareness and discuss challenges 2. Circulate company sustainability reports and policies
2. Lack of experience by Nestlé and suppliers	3. Standardise environmental data received from suppliers 4. Educate suppliers with webinars and short PowerPoint presentation 5. Develop concise and targeted LCI questionnaires 6. Provide 4–8 weeks to return completed LCI questionnaires
3. Lack of resources	7. Offer assistance to complete (remotely or physically present)
4. Identifying key actors within Nestlé and across the supply chain	8. Start building initial contact list from company network and expand 9. Search for environment/sustainability contacts on sustainability reports/websites
5. Engaging with actors with no direct business relationship	10. Contact people with direct business relationship to pursue request with indirect relationship 11. Integrate environmental considerations in the audit criteria of multi-tiered suppliers
6. Language barriers from non-UK data providers	12. Reduce communication to e-mails 13. Check with data providers preferred language 14. Provide translated questionnaire
7. Different technical language to express environmental, engineering and supply chain information	15. Align technical language to SI units/terminology
8. Commercial compromise	15. Anonymise data sources 16. Be transparent and clear on the application of data to reassure data provider on minimal compromise 17. Intermediary agent to host the data
9. Sensitivity of data disclosure	18. Aggregate data 19. Expand location point e.g. city to country
10. Confidentiality protection	20. Non-disclosure agreements (NDAs) 21. Respect environmental disclosure policy of suppliers
11. Conflict of interest with same person as data collector manager, provider and reviewer	22. Independently review data by third party
12. Navigating through a complex multi-tiered supplier systems	23. Engage with different suppliers and company supply chain/procurement function personnel to build common knowledge of supply chain structure
13. Visualising complexity	24. Create basic diagrams verified by supply chain actors
14. Modelling production processes	25. Engage with engineers to verify modelling

## Discussion

### Comparison with other data collection approaches

A critical assessment of a novel integrated LCI data collection process and strategy has been presented from the perspective of a multinational food company. It is the first of its kind and helps to fill an important gap in the existing knowledge on alternative strategies for LCI data collection. One of the key features of the process is the ability to leverage the resources of a manufacturing company to efficiently collect environmental data across the whole supply chain. For example, it was found that Nestlé—a multinational food company—was able to harness the perceived power of the organisation and translate into a supply chain leadership role to collect data. For example, the involvement of more than 50 different people across many divisions within the company was engaged to identify actors across the supply chain and to facilitate data exchange. In comparison to existing approaches (Ramos et al. 2016; Jungbluth et al. 2016; Recchioni et al. 2015; Mistry et al. 2016; Bellon-Maurel et al. 2014; Popp et al. 2013; Finkbeiner et al. 2003; Pomper 1998), the collection of data has been primarily on a voluntary basis where the implementation process is managed by a third party to drive the collection of data from different actors across the supply chain.

Another major benefit is the speed to collect primary data. The application of the LCI process at a confectionery factory in the UK found that a company-led approach was able to collect a portfolio of new environmental data in a relatively short period of 5 months. In total, 100 primary LCI datasets were collected from 67 ingredients and packaging suppliers across 13 countries. In comparison to other data collection approaches in the food industry (Ramos et al. 2016; Jungbluth et al. 2016; Milà i Canals et al. 2011), they do not provide an indication of the time involved to carry out data collection, especially for large amounts of data at different scales of problems, e.g. single product, multi-products, factory or even company level. Based on the experience gained, it is expected that a second round of data collection could result in a shorter timeframe of a few months. For example, with a projected timeframe of 3 months, this could result in 400 LCI datasets per year. Therefore, in addition to existing routes of data collection (Ramos et al. 2016; Mistry et al. 2016; Popp et al. 2013; Finkbeiner et al. 2003; Ecodesk 2015), the role of companies can significantly create more LCI data which can benefit both companies, supply chain actors and wider industry.

One other major benefit is the ability to create up-to-date and high-quality data. For example, the primary data collected has resulted in 100 new LCI datasets where the majority of the LCI datasets are not found anywhere in the literature (NREL 2014; Quantis 2014). In addition, the LCI datasets are relatively new where at the time of collecting, data were no more than 1 year old. Such data will be particularly useful for environmental analysis in the confectionery industry which in the EU alone comprises of over 11,000 confectionery manufacturers (CAOBISCO 2015).

Furthermore, another major benefit is the transparency in data collection to encourage high quality and reproducibility. For example, novel processes have been developed to visually describe the rationalisation and identification of ingredients, suppliers and customers across the supply chain compared to previous approaches (Ramos et al. 2016; Jungbluth et al. 2016; Recchioni et al. 2015; Mistry et al. 2016; Bellon-Maurel et al. 2014).

### Quality of data collected and gaps in data

The quality of data collected was found to vary considerably from both primary and secondary sources based on the pedigree matrix (Weidema and Wesnaes 1996). For example, the majority of primary data collected was found to be medium quality whereas the secondary data varied from high-to-low quality. However, the primary data had the potential to be high quality but was limited due to the representativeness criteria since the data only represented one site and not the whole market/country. Further statistical analysis showed the primary data had the lowest variation based on the standard deviations of DQS whereas secondary data for disposal and farm stage had the highest variation. For the retail and disposal level, only partially completed LCI questionnaires were returned. As such, data was sourced from the Ecoinvent database (Ecoinvent 2016). However, due to the generalised nature of LCI profiles in Ecoinvent, the quality was found to be low based on the ratings assigned on the pedigree matrix. Overall, the highest data quality was obtained for those companies that operate closer along the food supply chain to the multinational food company leading the data collection process. Hence, the critical stages of the supply chain requiring further research would be agricultural production (farm level) on one side, and retailers and waste treatment companies on the other side.

One of the major limitations found in practice was the collection of primary data at farm level. For the farm level, it was found few suppliers manage and operate vertically integrated operations from farm level to ingredient/packaging manufacture. However, due to the complex nature of farms, they can perform multiple services/functions over various periods of time creating multiple outputs. As such, the collection of primary data was out of scope as the timeframe to compile an inventory of all the materials and energy consumed at a farm level is much longer (e.g. months to years) compared to the other life cycle stages. In addition, the infrastructure and technology required to collect data is less advanced for farmers and have limited resources in terms of knowledge and expertise. Therefore, data was sourced from secondary LCI DBs such as WFLDB (Nemecek et al. 2014) and AgriBalyse (Koch and Salou 2013; Colomb et al. 2015). To this extent, the pursuit of specific farm level data should only be for significant raw materials as there is a trade-off between the volume of LCI data collected and resources expended in terms of people’s time. Despite the inclusion of secondary data, the LCI data collection process has shown that a multinational company can potentially engage and facilitate LCI data collection directly with farmers or indirectly through first-tier suppliers. Although, longer-term initiatives are required to establish environmental training through formal partnerships (e.g. TESCO 2015; Nestlé 2015a, b, c) to support and reduce the environmental impact at farms.

Overall, several recommendations are proposed to resolve data gaps and ensure the highest quality level for incomplete datasets, outdated data and data using proxies (Sadhukhan et al. 2014). For incomplete datasets, only sections which are completed and provide meaningful information are recommended. For outdated data, the pursuit of recent data is encouraged. For data using proxies, an investigation of the representation in terms of correlation and relevance should be assessed. In addition, the role of external LCA experts should be sought to provide additional quality assurances on the compiled dataset. For example, possible explanations of anomalies in data and verification of expected results.

### Effectiveness of tools and processes

An assessment of the different tools and processes applied during the LCI data collection is shown in Fig. 4. Although such tools and processes are found in the general literature (UNEP 2011; European Commission 2010; EPA 1993, 1995, 2014), we have presented for the first time an assessment of what was effective from the implementation experience on a two-axis graph showing the effectiveness to collect data with the effort required. It was found that the most effective process was the follow-up calls/e-mails with read receipts to data providers. However, such an approach was rather intensive and repetitive as records were kept to track communications.

Another effective process was the regular meetings with Nestlé personnel to review progress, identify any problems and provide support. In comparison, Ramos et al. (2016) found a web-based tool was effective to bring large numbers of companies together (e.g. 23 food small medium enterprises (SMEs)) on a single digital platform. Such a tool could be integrated within a company-led approach but would require an initial capital expenditure to develop.

### Challenges encountered during implementation

Over the course of the data collection process, several challenges were encountered as listed in Table 8. In comparison to the challenges found in the literature (UNEP 2011; European Commission 2010; EPA 2014), the major difference is the comprehensive overview with recommendations to resolve in the context of implementing a company-led LCI data collection process. For example, the data collected within the company, there was a major challenge of conflict of interest since the data collector manager, data provider and data exchange facilitator all work for the same company. Although this may be the case, the process to ensure robust datasets still remains by keeping records of data at different stages of transformation, validating data with experts in the company and pursuing data which is of high quality based on the pedigree data quality matrix. Further checks on the quality of data can be carried out by comparing data with similar materials/products and independently reviewing the data by a third party.

Another major challenge was the lack of engagement from supply chain actors, in particular ingredients and packaging suppliers. For example, a total of 45 of suppliers did not return the LCI questionnaires. Based on further discussion with suppliers, it was found that there were several reasons for either participating partially or not at all. For example, lack of resources and LCA experience, commercial compromise, sensitivity of data disclosure and confidentiality protection. It was found for the majority of suppliers, in particular the SMEs, that they did not have experience in completing a LCI questionnaire to a high level of detail where for some, it was completely new and for others, they had previously experienced multiple environmental data requests for various formats where their LCA teams employ LCA tools.

Furthermore, the commercial implications were a topic that came up often in the engagement process with suppliers both SME and large. Despite reassurance measures such as confidentiality protection through NDAs and anonymisation of data if shared in the public domain, the resistance to participate with some suppliers still remained. It was found that the level of participation depended largely on trust and relationships in terms of the people involved and the length of relationships.

### Motivations for companies to participate

Despite the challenges, several motivating factors were found for encouraging data providers to participate in the company-led LCI data collection process as part of their overarching Corporate Social Responsibility (CSR) strategy (Dahlsrud 2008). For example, the opportunity to collaborate with Nestlé (e.g. strengthen relationship, ways of working and partnerships), opportunity to learn about the environmental impact of their organisation/product in Nestlé’s products and opportunity to develop learning experience of LCI data request. However, in total, only 55% of suppliers returned the LCI questionnaire. A surprising finding was the lack of implementation from some companies who publically advocated sustainability improvements and supplier engagement both at an SME and (multinational corporation) MNC level as part of their CSR strategy. Despite this, the role of CSR can be a strong motivator for companies to participate as it was generally found that the sustainability commitments by different companies helped companies initially participate. As such, it is recommended that a range of initiatives are developed to encourage efficient LCI data collection by the company (i.e. Nestle). Such initiatives will aim to bring supply chain actors together to develop a mutual understanding on promoting sustainable supply chains. For example, workshops to discuss strategies to improve supply chain sustainability, specific partnerships with suppliers on key ingredients and LCA/environmental awareness training in the food industry.

### Towards a standardised procedure in the food sector

The LCI data collection process has the potential to transition towards a standardised procedure for the food sector subject to further application and consensus of stakeholders across the food industry. However, not all food companies have the ability to lead an LCI data collection process across the supply chain since these companies are either SMEs or do not manufacture finished products. As such, in this context, these companies can have a different role in which they can support organisations seeking to lead an LCI data collection process across the supply chain. Alternatively, such companies can group together to initiate an LCI data collection process for common materials shared between the companies. Despite this, the LCI data collection process and strategy provides an initial basis for other companies to further design their respective data collection strategies.

## Conclusions

This paper has presented a novel LCI data collection process developed, managed and implemented by a multinational food company. It represents one of the very first such studies of its type to critically assess the role and effectiveness of a multinational food company on collecting LCI data across the supply chain. For example, the application at a multi-product confectionery factory in the UK has resulted in a portfolio of 100 new environmental LCI datasets from the interaction with 67 ingredients and packaging suppliers across the globe and several food retailers. However, the majority of primary data collected was from ingredients and packaging suppliers, food factory and partial data for retailers and waste disposal providers with no data at the farm level. In addition, several challenges were encountered during implementation from the lack of experience, identifying key actors, confidentiality protection and complexity of multi-tiered supplier systems. Despite this, by using the internal resources, business relationships and influence of a multinational food company, it was found that a multinational company can play a critical role, especially in engagement and facilitation by transforming latent data found within companies or reported publically across the supply chain towards expansion of LCI data.

Furthermore, in order to encourage the reproducibility for other multinational companies, it is recommended the proposed LCI data collection process serves as a foundation to contribute towards a standardised procedure, in particular for food products. The specific features which can contribute towards a standardised procedure includes (1) process flow diagram of LCI data collection, (2) identification and role of actors in the company and across the supply chain, (3) supply chain maps, (4) processes to manage gaps in data and data quality and (5) LCI questionnaire. Overall, the key benefits of the proposed LCI data collection process includes (1) the ability to leverage the resources of a manufacturing company to efficiently collect environmental data across the whole supply chain, (2) the speed to collect primary data, (3) the ability to create up to date and medium to high quality data and (4) the increased transparency in data collection. However, further engagement with different food companies and applications across food categories would be required to develop a robust standardised procedure, especially supported by research institutes and NGOs.

## Electronic supplementary material

ESM 1 (DOCX 63 kb)

ESM 2 (XLSX 253 kb)

## References

[CR1] Agri-footprint gouda (2014) Agri-footprint methodology and basic principles version 1.0. http://www.agri-footprint.com/assets/Agri-Footprint-Part1-Methodologyandbasicprinciples-Version1.0.pdf. Accessed 02/03/2016

[CR2] ALCAS (2011) AusLCI datasets. http://alcas.asn.au/AusLCI/index.php/Datasets. Accessed 12/04/2015

[CR3] ALCAS (2014) Requirements for the development of AusLCI data sets. http://alcas.asn.au/AusLCI/Documents/AUSLCI_Requirements_30.pdf. Accessed 04/03/2015

[CR4] Almeida MI, Dias AC, Demertzi M, Arroja L (2015). Contribution to the development of product category rules for ceramic bricks. J Clean Prod.

[CR5] Ang CT, Morad N, Ismail N (2014). Challenges and possible drivers of LCA implementation in small and medium enterprises (SMEs) in Malaysia: a review. Pertanika J Soc Sci Hum.

[CR6] Bare J (2011). TRACI 2.0: the tool for the reduction and assessment of chemical and other environmental impacts 2.0. Clean Technol Environ.

[CR7] Bellon-Maurel V, Short MD, Roux P, Schulz M, Peters GM (2014). Streamlining life cycle inventory data generation in agriculture using traceability data and information and communication technologies—part I: concepts and technical basis. J Clean Prod.

[CR8] Berkhout F, Howes R (1997). The adoption of life-cycle approaches by industry: patterns and impacts. Resour Conserv Recy.

[CR9] BONSAI (2016) Big Open Network for Sustainability Assessment Information. https://bonsai.uno. Accessed 04/03/2015

[CR10] BSI PAS 2050 (2011) How to carbon your product footprint, identify hotspots and reduce your emission in the supply chain. In: The Guide to PAS2050–2011. http://shop.bsigroup.com/upload/shop/download/pas/pas2050.pdf. Accessed 28/09/2017

[CR11] CAOBISCO (2015) CAOBISCO annual report. http://caobisco.eu/public/images/page/caobisco-31032016102825-CAOBISCO-2015-Annual-Report-WEB.pdf. 05/03/2016

[CR12] Colomb V, Amar SA, Mens CB, Gac A, Gaillard G, Koch P, Mousset J, Salou T, Tailleur A, Werf HMG (2015) Oilseeds & fats crops and lipds. 22. D104

[CR13] Corporate Register (2017) Stats. http://www.corporateregister.com/stats. Accessed 30/04/2017

[CR14] Credit 360 (2015) About Us. http://www.credit360.com/credit/site/en/about_us.acds. Accessed 04/03/2016

[CR15] Dahlsrud A (2008). How corporate social responsibility is defined: an analysis of 37 definitions. Corp Soc Responsib Environ Manag.

[CR16] Dong YH, Ng T, Kwan AHK, Wu SK (2015). Substituting local data for overseas life cycle inventories – a case study of concrete products in Hong Kong. J Clean Prod.

[CR17] Dowson M, Grogan M, Birks T, Harrison D, Craig S (2012). Streamlined life cycle assessment of transparent silica aerogel made by supercritical drying. Appl Energ.

[CR18] EA (2014) National Packaging Waste Database. https://npwd.environment-agency.gov.uk/Public/PublicSummaryData.aspx. Accessed 04/03/2016

[CR19] Ecodesk (2015) About Us. https://www.ecodesk.com/aboutus. Accessed 04/03/2016

[CR20] Ecoinvent (2016) The ecoinvent database. http://www.ecoinvent.org/database/. Accessed 12/04/2016

[CR21] EPA (1993) Life-cycle assessment: inventory guidelines and principles. http://www.epa.gov/nscep/index.html. Accessed 04/03/2016

[CR22] EPA (1995) Guidelines for assessing the quality of life-cycle inventory analysis. http://www.epa.gov/nscep/index.html. Accessed 04/03/2015

[CR23] EPA (2014) Life cycle assessment (LCA). http://www.epa.gov/nrmrl/std/lca/lca.html. Accessed 04/03/2015

[CR24] European Commission (2008) Directive 2008/98/EC of the European Parliament and of the Council of 19 November 2008 on waste and repealing certain Directives. http://eur-lex.europa.eu/legal-content/EN/TXT/?uri=CELEX:32008L0098. Accessed 28/09/2017

[CR25] European Commission (2010) Joint Research Centre—Institute for Environment and Sustainability: International Reference Life Cycle Data System (ILCD) Handbook—general guide for life cycle assessment—detailed guidance. First edition March 2010. EUR 24708 EN. Luxembourg. Publications Office of the European Union

[CR26] European Commission (2013) Roadmap for the European Platform on life cycle assessment: facilitating data collection and sustainability assessments for policy and business. In: Fazio S, Recchioni M, Camillis C, Mathieux F, Pennington D, Allacker K, Ardente F, Benini L, Goralczyk M, Mancini L, Pant R, Sala S, Schau E. European Commission, Joint Research Centre, Institute for Environment and Sustainability. http://publications.jrc.ec.europa.eu/repository/bitstream/JRC85205/roadmap%20td%202%202%20%20awp%202013%20ensure_final.pd. Accessed 25/09/2017

[CR27] Finkbeiner M, Krinke S, Oschmann D, Saeglitz T, Schaper S, Schmidt W-P, Schnell R (2003). Data collection format for life cycle assessment of the German association of the automotive industry (VDA). Int J Life Cycle Assess.

[CR28] Finnveden G, Hauschild MZ, Ekvall T, Guinee J, Heijungs R, Hellweg S, Koehler A, Pennington D, Suh S (2009). Recent developments in life cycle assessment. J Environ Manag.

[CR29] Goedkoop M, Heijungs R, Huijbregts M, de Schryver A, Struijs J, van Zelm R (2009). ReCiPe 2008. A life assessment method which comprises harmonised category indicators at the midpoint and the endpoint level. Report I: characterisation.

[CR30] Guinée JB, Gorrée M, Heijungs R, Huppes G, Kleijn R, de Koning A, van Oers L, Wegener Sleeswijk A, Suh S, Udo de Haes HA, de Bruijn H, van Duin R, Huijbregts MAJ (2002). Handbook on life cycle assessment. Operational guide to the ISO standards.

[CR31] IPCC (2006) IPCC guidelines for National Greenhouse Gas Inventories. Intergovernmental panel on climate change, Geneva

[CR32] Islam S, Ponnambalam SG, Lam HL (2016). Review on life cycle inventory: methods, examples and applications. J Clean Prod Part B.

[CR33] ISO (2006) ISO 14044, environmental management—life cycle assessment—requirements and guidelines, Geneva

[CR34] Jimenez-Gonzalez C, Kim S, Overcash M (2000). Methodology for developing gate-to-gate life cycle inventory information. Int J Life Cycle Assess.

[CR35] Jolliet O, Saade-Sbeih M, Shaked S (2015). Environmental life cycle assessment.

[CR36] Jungbluth N, Keller R, König A (2016). ONE TWO WE—life cycle management in canteens together with suppliers, customers and guests. Int J Life Cycle Assess.

[CR37] Kellens K, Dewulf W, Overcash M, Hauschild M, Duflou JR (2011). Methodology for systematic analysis and improvement of manufacturing unit process life cycle inventory (UPLCI) part 1: methodology description. Int J Life Cycle Assess.

[CR38] Kim DB, Shin S-J, Shao G, Brodsky A (2015). A decision-guidance framework for sustainability performance analysis of manufacturing processes. Int J Adv Manuf Tech.

[CR39] Koch P, Salou T (2013) Agribalyse: methodology version 1.1. http://www.ademe.fr/sites/default/files/assets/documents/agribalyse_methodology_report_v1.1.pdf. Accessed: 01/03/2015

[CR40] Lesage P, Samson R (2016). The Quebec life cycle inventory database project—using the ecoinvent database to generate, review, integrate, and host regional LCI data. Int J Life Cycle Assess.

[CR41] Meinrenken CJ, Sauerhaft BC, Garvan AN, Lackner KS (2014). Combining life cycle assessment with data science to inform portfolio-level value-chain engineering—a case study at PepsiCo Inc. J Ind Ecol.

[CR42] Miah JH, Griffiths A, McNeill R, Poonaji I, Martin R, Morse S, Yang A, Sadhukhan J (2015). Creating an environmentally sustainable food factory: a case study of the lighthouse project at Nestlé. Procedia CIRP.

[CR43] Miah JH, Griffiths A, McNeill R, Poonaji I, Martin R, Morse S, Yang A, Sadhukhan J (2015). A small-scale transdisciplinary process to maximising the energy efficiency of food factories: insights and recommendations from the development of a novel heat integration framework. Sustain Sci.

[CR44] Mila i Canals L, Sim S, Garcia-Suarez T, Neuer G, Herstein K, Kerr C, Rigarlsford G, King H (2011). Estimating the greenhouse gas footprint of Knorr. Int J Life Cycle Assess.

[CR45] Mistry M, Gediga J, Boonzaier S (2016). Life cycle assessment of nickel products. Int J Life Cycle Assess.

[CR46] Nemecek T, Bengoa X, Lansche J, Mouron P, Rossi V, Humbert S (2014). Methodological guidelines for the life cycle inventory of agricultural products. Version 2.0, July 2014.

[CR47] Nestlé (2013) Insight: how we’re further building sustainability into our product design process. http://www.nestle.com/media/newsandfeatures/ecodex-insight-blog. Accessed 04/03/2016

[CR48] Nestlé (2015a) Nestlé in society (full report). http://www.nestle.com/asset-library/documents/library/documents/corporate_social_responsibility/nestle-csv-full-report-2014-en.pdf. Accessed 12/05/2016

[CR49] Nestlé (2015b) The Nestlé Cocoal Plan. http://www.nestlecocoaplan.com/. Accessed 12/12/2016

[CR50] Nestlé (2015c) The Nescafe Plan. http://www.nescafe.com/sustainability_en_com.axcms. Accessed 04/03/2016

[CR51] Ning S-K, Chang N-B, Hung M-C (2013). Comparative streamlined life cycle assessment for two types of municipal solid waste incinerator. J Clean Prod.

[CR52] NREL (2014) Data Discovery. https://www.lcacommons.gov/nrel/search. Accessed 04/03/2016

[CR53] PEF World Forum (2013) Product Environmental Footprint (PEF) Guide. http://www.pef-world-forum.org/wp-content/uploads/2013/10/PEF_Guide.pdf. Accessed 12/04/2016

[CR54] Pennington D, Wolf MA, Bersani R, Pretato U (2007). Overcoming barriers to the broader implementation of life cycle thinking in business and public administration. Int J Life Cycle Asses.

[CR55] PlasticsEurope (2015) Eco-profile. http://www.plasticseurope.org/plasticssustainability/eco-profiles.aspx. Accessed 12/04/2016

[CR56] Pomper SD (1998) Managing LCI data gathering. SAE technical paper series, p 1–6

[CR57] Popp JS, Thoma GJ, Mulhern J, Jaeger A, LeFranc L, Kemper N (2013). Collecting comprehensive farm level data through a collaborative approach: a framework developed for a life cycle assessment of fluid milk production in the US. Int Dairy J.

[CR58] Quantis (2014) World Food LCA Database. http://www.quantis-intl.com/wfldb/. Accessed 04/03/2016

[CR59] Ramos S, Larrinaga L, Albinarrate U, Jungbluth N, Ingolfsdottir GM, Yngvadottir E, Landquist B, Woodhousee A, Olafsdottir G, Esturo A, Zufía J, Perez-Villareal B (2016). SENSE tool: easy-to-use web-based tool to calculate food product environmental impact. Int J Life Cycle Assess.

[CR60] Rebitzer G, Buxmann K (2005). The role and implementation of LCA within life cycle management at Alcan. J Clean Prod.

[CR61] Rebitzer G, Ekvall T, Frischknecht R, Hunkler D, Norris G, Rydberg T, Schmidt W-P, Suh S, Weidema BP, Pennington DW (2004). Life cycle assessment. Part 1: framework, goal and scope definition, inventory analysis, and applications. Environ Int.

[CR62] Recchioni M, Blengini GA, Fazio S, Mathieux F, Pennington D (2015). Challenges and opportunities for web-shared publication of quality-assured life cycle data: the contributions of the life cycle data network. Int J Life Cycle Assess.

[CR63] Resta B, Gaiardelli P, Pinto R, Dotti S (2016). Enhancing environmental management in the textile sector: an organisational-life cycle assessment approach. J Clean Prod.

[CR64] Roches A, Nemecek T, Gailard G, Plassmann K, Sim S, King H, Canals LM (2010). MEXALCA: a modular method for the extrapolation of crop LCA. Int J Life Cycle Assess.

[CR65] Sadhukhan J, Ng KS, Hernandez EM (2014). Biorefineries and chemical processes. Design, integration and sustainability analysis.

[CR66] Scanlon KA, Cammarata C, Siart S (2013). Introducing a streamlined life cycle assessment approach for evaluating sustainability in defense acquisitions. Environ Syst Decis.

[CR67] Skone TJ, Curran MA (2005). LCAccess—global directory of LCI resources. J Clean Prod.

[CR68] Spencer S, Kneebone M (2007) FoodMap: a comparative analysis of Australian food distribution channels. http://www.agriculture.gov.au/SiteCollectionDocuments/ag-food/food/national-food-plan/submissions-received/foodmap-an-analysis-of-the-australian-food-supply-chain-30-july.pdf. Accessed 04/03/2016

[CR69] Spencer S,Kneebone M (2012) FOODmap: an analysis of the Australian food supply chain. http://www.agriculture.gov.au/SiteCollectionDocuments/ag-food/food/national-food-plan/submissions-received/foodmap-an-analysis-of-the-australian-food-supply-chain-30-july.pdf. Accessed 04/03/2016

[CR70] Stoessel F, Juraske R, Pfsiter S, Hellweg S (2012). Life cycle inventory and carbon and water foodprint of fruits and vegetables: application to a swiss retailer. Environ Sci Technol.

[CR71] Suh S, Huppes G (2005). Methods for life cycle inventory of a product. J Clean Prod.

[CR72] TESCO (2015) Milk & cream. http://realfood.tesco.com/our-food/milk/our-milk.html. Accessed 04/03/2016

[CR73] Testa F, Nucci B, Tessitore S, Iraldo F, Daddi T (2016). Perceptions on LCA implementation: evidence from a survey on adopters and nonadopters in Italy. Int J Life Cycle Assess.

[CR74] UNEP (2011) Global guidance principles for life cycle assessment databases—a basis for Greener Processes and Products. http://www.unep.org/pdf/Global-Guidance-Principles-for-LCA.pdf. Accessed 04/03/2016

[CR75] Weidema BP, Wesnaes MS (1996). Data quality management for life cycle inventories—an example of using data quality indicators. J Clean Prod.

[CR76] Weidema BP, Bauer Ch, Hischier R, Mutel Ch, Nemecek T, Reinhard J, Vadenbo CO, Wernet G (2013) The ecoinvent database: overview and methodology, data quality guideline for the ecoinvent database version 3. www.Ecoinvent.Org. Accessed 20/05/2016

[CR77] WFLDB (2014) Methodological guidelines for the life cycle inventory of agricultural products. http://www.quantis-intl.com/files/2714/0626/8848/WFLDB_MethodologicalGuidelines_20140723_v2.0.pdf. Accessed 04/03/2016

