# Supplementary material for: A framework for increasing the availability of life cycle inventory data based on the role of multinational companies
Source: Int J Life Cycle Assess. 2017 Oct 4;23(9):1744–60. doi: 10.1007/s11367-017-1391-y (PMC6428398; doi:10.1007/s11367-017-1391-y)
Supplement: Supplementary file 1 — (DOCX 63 kb) [file 11367_2017_1391_MOESM1_ESM.docx]

Electronic supplementary material

[Data availability, data quality](javascript:popupClassificationDetail(8))

**A framework for increasing the availability of life cycle inventory data based on the role of multinational companies**

**Jamal Hussain Miah^1,2^ • Andrew Griffiths^3^ • Ryan McNeill^4^ • Sharla Halvorson^5^ • Urs Schenker^6^ • Namy Espinoza-Orias^6^ • Stephen Morse^2^ • Aidong Yang^7^ • Jhuma Sadhukhan^2^**

Received: 20 December 2016 / Accepted: 22 August 2017

© Springer-Verlag Berlin Heidelberg 2017

Responsible editor: Niels Jungbluth

^1^ Nestlé UK Ltd, Rowan Drive, Fawdon, Newcastle Upon Tyne, NE3 3TR, UK

^2^ Centre for Environment and Sustainability (CES), Faculty of Engineering & Physical Sciences, University of Surrey, Guildford, GU2 7XH, UK

^3^ Nestlé UK Ltd, Group Technical and Production, Haxby Road, York, YO91 1XlY, UK

^4^ Nestlé Confectionery Product & Technology Centre (PTC), Haxby Road, York, YO91 1XY, UK

^5^ Nestlé Research Centre (NRC), CT-Nutrition, Health, Wellness & Sustainability, 1000 Lausanne 26, Switzerland

^6^Nestlé Research Centre (NRC), Sustainability & Novel Packaging, 1000 Lausanne 26, Switzerland

^7^ Department of Engineering Science, University of Oxford, Parks Road, Oxford, OX1 3PJ, UK

🖂 Jamal Hussain Miah

[j.miah@surrey.ac.uk](mailto:j.miah@surrey.ac.uk)

| **1. Supplier information** |
| --- |
| **Below is a list of people who may be able to contribute to the completion of this section:**   \| 1. **Key account manager** 2. **Environment / sustainability manager** 3. **Nestlé LCA contact** \| 1. **Factory manager** \| \| --- \| --- \| |

If the information below is incorrect please change accordingly.

| **1.1. Supplier name** |  |
| --- | --- |
| **1.2. Key account manager name** |  |
| **1.3. Key account manager email address** |  |
| **1.4. Environment / sustainability manager name** |  |
| **1.5. Environment / sustainability manager email address** |  |
| **1.6. Supplier manufacturing site address** |  |
| **1.7. Manufacturing site vendor number** |  |
| **1.8. Name of purchased supplied product** |  |
| **1.9. Material code of purchased supplied product** |  |

**2.0. In Figure 1 what stages of the supply chain can you provide data?** If stage 1 does not fall into direct operations but can be collected through further engagement please mark the box.

| Stage 1:  Raw Materials  Extraction |  | Stage 2:  Ingredients  Manufacturing Site |  | Stage 3:  Ingredients  Distribution Centre |  |
| --- | --- | --- | --- | --- | --- |

**2.1. If stage 1 was selected can you request environmental information if a data template was provided, subject to further discussion?**

| YES |  | NO |  |
| --- | --- | --- | --- |

**2.2. Is there a Life Cycle Assessment available for the supplied product?** If yes, would you be able to provide a copy of the LCA for the supplied product subject to further discussion.

| YES |  | NO |  |
| --- | --- | --- | --- |

**2.3. Is the manufacturing site of the supplied product ISO14000 certified?**

| YES |  | NO |  |
| --- | --- | --- | --- |

**2.4. Are you classified as an SME?** Please see definition of [SME by European Commission](http://ec.europa.eu/enterprise/policies/sme/facts-figures-analysis/sme-definition/index_en.htm).

| YES |  | NO |  |
| --- | --- | --- | --- |

**N.B. The information requested for the rest of this form applies only to stage 2: Ingredients manufacturing site.**

| **2. Production** |
| --- |
| **Below is a list of people who may be able to contribute to the completion of this section:**   \| 1. **Factory production staff** 2. **Environment / sustainability manager** 3. **Factory manager** \| 1. **Factory engineers** \| \| --- \| --- \| |

**2.1. Are there multiple factories at the manufacturing site?** If yes, please indicate how many.

| YES |  | NO |  |
| --- | --- | --- | --- |
| Number of factories |  |  |  |

**2.2. What is the production volume of the different products at the manufacturing site?** Please complete the table.

| Factory | Product name | Quantity | Units | Data coverage | Data source |
| --- | --- | --- | --- | --- | --- |
|  |  |  |  | 2013 |  |
|  |  |  |  | 2013 |  |
|  |  |  |  | 2013 |  |
|  |  |  |  | 2013 |  |
|  |  |  |  | 2013 |  |
|  |  |  |  | 2013 |  |
|  |  |  |  | 2013 |  |
|  |  |  |  | 2013 |  |
|  |  |  |  | 2013 |  |

*Please insert new rows as necessary.*

**2.3. Can you please provide a block diagram of the supplied product process?** If yes, please provide the block diagram as an attachment.

| YES |  | NO |  |
| --- | --- | --- | --- |

**2.4. What are the raw materials required to manufacture 1 kg or 1 ton of supplied product?** Please complete the table.

| Raw material name | Quantity | Units | Data coverage | Data source |
| --- | --- | --- | --- | --- |
|  |  |  | 2013 |  |
|  |  |  | 2013 |  |
|  |  |  | 2013 |  |
|  |  |  | 2013 |  |
|  |  |  | 2013 |  |
|  |  |  | 2013 |  |
|  |  |  | 2013 |  |
|  |  |  | 2013 |  |
|  |  |  | 2013 |  |

*Please insert new rows as necessary.*

| **3. Land footprint** |
| --- |
| **Below is a list of people of may be able to contribute to the completion of this section:**   \| 1. **Environment / sustainability manager** 2. **Factory manager** 3. **Factory engineers** \| 1. **Facilities manager** 2. **Factory SHE manager** \| \| --- \| --- \| |

**3.1. What is the land area covered by the supplied product manufacturing plant(s)?** Please provide data in m^2^.

|  | **m^2^** |
| --- | --- |

**3.2. What is the land area covered by the total manufacturing site?** Please provide data in m^2^.

|  | **m^2^** |  |  |
| --- | --- | --- | --- |
| **4. Energy** | | | |
| **Below is a list of people of may be able to contribute to the completion of this section:**   \| 1. **Environment / sustainability manager** 2. **Factory manager** 3. **Factory engineers** \| 1. **Facilities manager** 2. **Factory SHE manager** \| \| --- \| --- \| | | | |

**4.1. At what spatial level can you provide energy data?** If calculations and approximations are required please mark the relevant boxes.

| Product level |  | Plant level |  | Factory level |  | Manufacturing site level |  |
| --- | --- | --- | --- | --- | --- | --- | --- |

**4.2. Please provide the following energy data at a factory level.**

| Energy type | Quantity | Units | Data coverage | Data source |
| --- | --- | --- | --- | --- |
| Electricity Import |  |  | 2013 |  |
| Electricity Export |  |  | 2013 |  |
| Natural Gas |  |  | 2013 |  |
| Steam |  |  | 2013 |  |
| Crude oil |  |  | 2013 |  |
| Coal |  |  | 2013 |  |
| Other  (please specify) |  |  | 2013 |  |

*Please insert new rows as necessary.*

**4.3. Please provide the following energy data for the supplied product level.** If approximations are made, please make clear.

| Energy type | Quantity | Units | Data coverage | Data source |
| --- | --- | --- | --- | --- |
| Electricity Import |  |  | 2013 |  |
| Electricity Export |  |  | 2013 |  |
| Natural Gas |  |  | 2013 |  |
| Steam |  |  | 2013 |  |
| Crude oil |  |  | 2013 |  |
| Coal |  |  | 2013 |  |
| Other  (please specify) |  |  | 2013 |  |

*Please insert new rows as necessary.*

**4.4. Please complete the table about the imported electricity?**

| Country | Quantity | Units | Data coverage | Data source |
| --- | --- | --- | --- | --- |
|  |  |  | 2013 |  |
|  |  |  | 2013 |  |

*Please insert new rows as necessary.*

**4.5. Is there combustion equipment on-site that supplies energy to the processes manufacturing the supplied product?** If yes, please complete the table and state if it is a gas boiler, coal boiler or CHP.

| YES |  | NO |  |  | Gas boiler |  |  | Coal boiler |  |  | CHP |  |
| --- | --- | --- | --- | --- | --- | --- | --- | --- | --- | --- | --- | --- |

| Emission type | Quantity | Units | Data coverage | Data source |
| --- | --- | --- | --- | --- |
| CO_2_ |  |  | 2013 |  |
| CH_4_ |  |  | 2013 |  |
| N_2_O |  |  | 2013 |  |
| Other  (please specify) |  |  | 2013 |  |

*Please insert new rows as necessary.*

**4.6. What is the Net Calorific Value of the gas fuel type?** Please provide a reference.

|  | **MJ/kg** |  |  |
| --- | --- | --- | --- |
| **5. Water** | | | |
| **Below is a list of people of may be able to contribute to the completion of this section:**   \| 1. **Environment / sustainability manager** 2. **Factory manager** 3. **Factory engineers** \| 1. **Facilities manager** 2. **Factory SHE manager** \| \| --- \| --- \| | | | |

**5.1. Is water used in the manufacturing process of the product supplied?** If yes, please complete the rest of this section.

| YES |  | NO |  |
| --- | --- | --- | --- |

**5.2. At what spatial level can you provide water data?**

| Product level |  | Plant level |  | Factory level |  | Manufacturing site level |  |
| --- | --- | --- | --- | --- | --- | --- | --- |

**5.3. Please provide the following water data at a factory level.**

| Source | Quantity | Units | Data coverage | Data source |
| --- | --- | --- | --- | --- |
| Mains water |  |  | 2013 |  |
| River water |  |  | 2013 |  |
| Ground water |  |  | 2013 |  |
| Recycled water |  |  | 2013 |  |
| Other  (please specify) |  |  | 2013 |  |

*Please insert new rows as necessary.*

**5.4. Please provide the following water data for the supplied product level.** If approximations are made, please make clear.

| Source | Quantity | Units | Data coverage | Data source |
| --- | --- | --- | --- | --- |
| Mains water |  |  | 2013 |  |
| River water |  |  | 2013 |  |
| Ground water |  |  | 2013 |  |
| Recycled water |  |  | 2013 |  |
| Other  (please specify) |  |  | 2013 |  |

*Please insert new rows as necessary.*

| **6. Atmospheric Emissions from processes** |
| --- |

**Below is a list of people of may be able to contribute to the completion of this section:**

| 1. **Environment / sustainability manager** 2. **Factory manager** 3. **Factory engineers** | 1. **Facilities manager** 2. **Factory SHE manager** |
| --- | --- |

**6.1. Are there emissions from the processes of the manufactured product?** If yes, please complete the table.

| YES |  | NO |  |
| --- | --- | --- | --- |

| Emission type | Equipment source | Quantity | Units | Data coverage | Data source |
| --- | --- | --- | --- | --- | --- |
| Particulate matter |  |  |  | 2013 |  |
| VOCs |  |  |  | 2013 |  |
| Water vapour |  |  |  | 2013 |  |
| Other  (please specify) |  |  |  | 2013 |  |

*Please insert new rows as necessary.*

| **7. Solid waste from processes** |
| --- |

**Below is a list of people of may be able to contribute to the completion of this section:**

| 1. **Environment / sustainability manager** 2. **Factory manager** 3. **Factory engineers** | 1. **Facilities manager** 2. **Factory SHE manager** |
| --- | --- |

**7.1. At what spatial level can you provide solid waste data?**

| Product level |  | Plant level |  | Factory level |  | Manufacturing site level |  |
| --- | --- | --- | --- | --- | --- | --- | --- |

**7.2. Are there solid wastes generated from the processes of the manufactured product?** If yes, please complete the table.

| YES |  | NO | |  |  |  |  |  |  |
| --- | --- | --- | --- | --- | --- | --- | --- | --- | --- |
| Solid waste type | | | | Quantity | | | Units | Data coverage | Data source |
|  | | | |  | | |  | 2013 |  |
|  | | | |  | | |  | 2013 |  |
|  | | | |  | | |  | 2013 |  |
|  | | | |  | | |  | 2013 |  |
|  | | | |  | | |  | 2013 |  |
|  | | | |  | | |  | 2013 |  |
|  | | | |  | | |  | 2013 |  |

*Please insert new rows as necessary.*

| **8. Liquid waste from processes** |
| --- |

**Below is a list of people of may be able to contribute to the completion of this section:**

| 1. **Environment / sustainability manager** 2. **Factory manager** 3. **Factory engineers** | 1. **Facilities manager** 2. **Factory SHE manager** |
| --- | --- |

**8.1. At what spatial level can you provide wastewater data?**

| Product level |  | Plant level |  | Factory level |  | Manufacturing site level |  |
| --- | --- | --- | --- | --- | --- | --- | --- |

**8.2. Are there liquid wastes generated from the processes of the manufactured product?** If yes, please complete the table.

| YES |  | NO |  |
| --- | --- | --- | --- |

| liquid waste type | Quantity | Units | COD (mg/l) | BOD (mg/l) | Data coverage | Data source |
| --- | --- | --- | --- | --- | --- | --- |
| Process wastewater |  | m^3^ |  |  | 2013 |  |
|  |  |  |  |  | 2013 |  |
|  |  |  |  |  | 2013 |  |

*Please insert new rows as necessary.*

| **9. Transportation of supplied product to York factory** |
| --- |

**Below is a list of people of may be able to contribute to the completion of this section:**

| 1. **Key account manager** 2. **Logistics manager** |  |
| --- | --- |

**9.1. Can you please describe the transportation route from the supplied product manufacturing site to York factory?** If possible, please breakdown the transport route into steps.

Example

| Start location | End location | Distance | Units | Mode of transport | Percentage of vehicle filling (%) on **supply** journey | Percentage of vehicle filling (%) on **return** journey |
| --- | --- | --- | --- | --- | --- | --- |
| Sugar Corp Factory  123 Sugar Lane  USA  DX1 5FI | Sugar Corp Distribution Centre | 100 | km | Truck | 50% | 0% |
| Sugar Corp Distribution Centre | East Cost Shipping Port | 200 | km | Truck | 5% | 0% |
| East Cost Shipping Port | Hull Shipping Port | 5000 | km | Ship | Unknown | 0% |
| Hull Shipping Port | Supplier Distribution Centre | 150 | km | Truck | Unknown | 0% |
| Supplier Distribution Centre | Fawdon factory | 100 | km | Truck | Unknown | 100% (empty containers and pallets) |
|  |  |  |  |  |  |  |

To be completed by supplier.

| Start location | End location | Distance | Units | Mode of transport | Percentage of vehicle filling (%) on **supply** journey | Percentage of vehicle filling (%) on **return** journey |
| --- | --- | --- | --- | --- | --- | --- |
|  |  |  |  |  |  |  |
|  |  |  |  |  |  |  |
|  |  |  |  |  |  |  |
|  |  |  |  |  |  |  |
|  |  |  |  |  |  |  |
|  |  |  |  |  |  |  |

*Please insert new rows as necessary.*
